# Monozygotic twin sisters discordant for familial hemiplegic migraine

**DOI:** 10.1186/1129-2377-14-77

**Published:** 2013-09-16

**Authors:** José Barros, Rui Barreto, Ana Filipa Brandão, Joana Domingos, Joana Damásio, Cristina Ramos, Carolina Lemos, Jorge Sequeiros, Isabel Alonso, José Pereira-Monteiro

**Affiliations:** 1 Serviço de Neurologia, Departamento de Neurociências, Hospital de Santo António (HSA), Centro Hospitalar do Porto (CHP), Largo Prof. Abel Salazar, 4099-001 Porto, Portugal; 2 Instituto de Ciências Biomédicas Abel Salazar (ICBAS), Universidade do Porto, Porto, Portugal; 3 Centro Hospitalar de Entre Douro e Vouga (CHEDV), Santa Maria da Feira, Portugal; 4 Instituto de Biologia Molecular Celular (IBMC), Universidade do Porto, Porto, Portugal

**Keywords:** Hemiplegic migraine, Migraine with typical aura, Monozygotic twins, Forced thinking

## Abstract

**Background:**

The high concordance rate of migraine in monozygotic twin pairs has long been recognised. In the current study, we present a monozygotic twin pair discordant for familial hemiplegic migraine (FHM).

**Case presentations:**

We evaluated 12 adult family members in 2012. The twin pair was separately examined by neurologists at different time points. Mutation screening was performed for known FHM-related genes. The monozygosity of the twins was verified. Eleven individuals had a history of migraine or paroxysmal neurological symptoms, including four patients with motor aura. No mutations were detected in the *CACNA1A, ATP1A2*, *SCN1A*, *PRRT2* or *NOTCH3* genes. The monozygotic twin sisters, aged 52, were discordant for age of onset, motor aura and neuropsychological aura (forced thinking). Overall, the family members presented a wide range of phenotypical features.

**Conclusions:**

Familial hemiplegic migraine is a monogenic disorder that is distinct from migraine with typical aura. However, in certain families with motor aura, such as this one, it is possible that the most severe phenotype is caused by an unlikely combination of polygenic traits and non-genetic factors. In these kindreds, we propose that hemiplegic aura is only a severe and complex form of typical aura.

## Background

Migraine is a genetic disease, [1,2] although environmental factors are important in its aetiology. The probandwise and pairwise concordance rates of migraine are significantly higher in monozygotic compared with dizygotic twin pairs [3-6]. A recent meta-analysis has demonstrated a heritability of approximately 50% for migraine [7].

The genes identified for familial hemiplegic migraine (FHM) provide the strongest argument in favour of a genetic background. FHM is a rare type of autosomal dominant migraine with aura (MWA). Diagnostic criteria require motor aura associated with at least one other aura symptom and the presence of identical episodes in at least one first- or second-degree relative [8]. The three types of FHM are associated with mutations in different ion channel genes (*CACNA1A*, *ATP1A2,* and *SCN1A)*[8]. These mutations are only observed in a minority of patients and families, [9,10] suggesting the presence of mutations in additional genes, which remain unknown at present. Recently, mutations in the proline-rich transmembrane protein 2 gene (*PRRT2*) have been shown to be associated with hemiplegic migraine [11].

To our knowledge, only two studies on FHM involving pairs of monozygotic twins have been published. In 1995, Ducros et al. reported a pair of 42-year-old twin sisters discordant for pure FHM [12]. Recently, Castiglioni et al. have described a pair of monozygotic twins with identical FHM and paroxysmal kinesigenic dyskinesia due to a *PRRT2* mutation [13]*.*

In the current study, we describe a family whose members presented with different types of migraine transmitted as an autosomal dominant trait. However, a pair of monozygotic twins exhibited discordance for familial hemiplegic migraine.

## Case presentations

**Figure 1:**
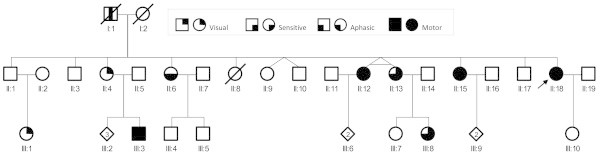
**Pedigree structure.** Black-filled symbols indicate hemiplegic migraine; black-filled quadrants, non-motor aura symptoms; vertical bar, migraine without aura; empty symbols, unaffected members; arrow, proband; squares, males; circles, females; and diagonal lines, deceased.

After examining a woman (II:18) who presented with FHM, we evaluated 12 of her adult family members in 2012 (Figure 1). Two monozygotic twins (II:12, II:13; age: 52 years) were separately examined by different neurologists at different time points and were subjected to 3-tesla brain MRI on the same date. The medical records of deceased family members (I:1, II:8) were reviewed. The pedigrees were progressively improved based upon statements from the patients and their relatives.

The present study was approved by the Ethics Committee of the Hospital de Santo António.

DNA was obtained from the peripheral blood of the affected and healthy family members after acquiring written informed consent. Samples were stored at the authorised biobank at Instituto de Biologia Molecular e Celular.

Mutation screening was performed for known FHM-related genes. The genetic study was performed by PCR amplification followed by direct bidirectional sequencing of the entire coding regions and intronic flanking sequences of the *CACNA1A, ATP1A2*, *SCN1A*, *PRRT2* and *NOTCH3* genes. Large gene rearrangements in *CACNA1A, ATP1A2* and *SCN1A* were screened by multiplex ligation-dependent probe amplification. Monozygosity of the twins was confirmed using an identification panel consisting of 15 polymorphic markers plus amelogenin (ESSplex Kit, Qiagen)*.*

The clinical characteristics of the presently described family are summarised in Table 1 and Figure 1. The patriarch, six of his 12 children and 4 of his 14 grandchildren had a history of migraine headache or paroxysmal neurological symptoms.

**Table 1:** Main clinical features of the family members

**Subject gender/age (yrs)**	**Onset, remission, (peak) (yrs)**	**Lifetime occurrence (no. episodes)**	**Aura**				**Headache**			**Accompanying symptoms**	**Precipitating factors**
			**Duration (mins)**	**Type**	**Location**	**Laterality**	**Duration (hrs)**	**Character/ Intensity**	**Laterality**		
II:4 F/63	12	>50	5	Flickering light	-	Bilateral	4	Throbbing/ Severe	Left	Pn, Pt	Stress
II:6 F/57	13	1	30	Aphasic; sensitive; perceived limb shape distortions	F, UL	Right	-	-	-	-	-
II:12 F/52	11, 48	>20	10	Flickering light; forced thinking; sensitive; aphasic; motor	F, UL	Side-shifting or bilateral	48	Throbbing/ Severe	Right	N, V, Pn, Pt	Menstruation; stress; minor head trauma
II:13 F/52	22 (45)	>50	20	Scintillating scotoma; sensitive; aphasic	F, T, UL	Side-shifting or bilateral	48	Throbbing/ Severe	Side-shifting or bilateral	N, V, Pn, Pt	Hysterectomy
II:15 F/50	18, 47	>50	20	Flickering light; sensitive; motor; dizziness	F, T, UL	Side-shifting	12-48	Throbbing/ Severe	Right	N, V, Pn, Pt	Menstruation; cheese; chocolate; Insomnia
II:18 F/45	18	>50	15	Scintillating scotoma; sensitive; aphasic; motor; dizziness	F, UL	Side-shifting	48	Throbbing/ Severe	Bilateral	N, V, Pn, Pt	Menstruation
III:1 F/37	15	>20	5-10	Scintillating scotoma	-	Side-shifting	12	Throbbing/ Severe	Bilateral	N, V, Pn, Pt	-
III:3 M/25	13 (20)	>20	30	Scintillating scotoma; sensitive; aphasic; motor	UL	Right	16	Throbbing/ Severe	Side-shifting	N, V, Pn, Pt	Minor head trauma
III:4 M/28	7, 12	>20	-	Dizziness	-	-	16	Throbbing/ Severe	Side-shifting	N, Pn, Pt	Stress
III:8 F/27	13	>50	20	Flickering light; sensitive; aphasic	F, UL	Side-shifting or bilateral	24-48	Throbbing/ Severe	Side-shifting (80% right)	N, V, Pn, Pt	Pregnancy; stress

F, face; T, tongue; UL, upper limb; LL, lower limb; Tr, trunk; N, nausea; V, vomiting; Pt, photophobia; Pn, phonophobia; S, somnolence

Four patients presented with motor aura (the motor aura diagnosis was based on patients’ and relatives’ descriptions). We did not observe any of the patients during motor aura. The other patients had auras of various types either alone or in combination. All family members who were diagnosed with MWA or FHM also experienced episodes of migraine without aura (MoA). Neurologic examinations were normal for all patients. No mutations were found in the *CACNA1A, ATP1A2, SCN1A*, *PRRT2* or *NOTCH3* genes. Additionally, large gene rearrangements in *CACNA1A, ATP1A2* and *SCN1A* were ruled out.

### Clinical features of the twin pair

The monozygotic twin sisters, currently age 52, were physically indistinguishable during childhood and had been raised in different family environments since the age of 11. Monozygosity was confirmed by the concordance of the alleles in both individuals for all 15 polymorphic markers tested.

At menarche, one of the twins experienced the first paroxysmal neurological episode, followed by right-sided unilateral headache. These episodes began with side-shifting scintillating scotoma for 5 minutes, followed by tingling and numbness of both hands, progressing toward the elbows and eventually reaching the face, cheek and tongue. The episodes lasted approximately 30 minutes. Manipulation of objects during the episodes was imperfect. Unilateral headache began during the aura and was associated with severe vomiting. The symptoms disappeared during sleep. The patient never sought medical care during the acute phase. The frequency of complex episodes increased with age, reaching a peak of two episodes per week during her third pregnancy at age 25. From that point on, motor aphasia occurred during and after the somatosensory symptoms. Rapid and repetitive sequences of compulsive thoughts coincided with the aphasia; these thoughts were unavoidable, strange and extremely detailed. The themes were always religious, including monasteries, churches, biblical characters, faces of clergy, liturgical garments and objects of Catholic worship. These thought sequences lasted approximately 10 minutes and never occurred in isolation. In one in five episodes, the patient simultaneously experienced side-shifting weakness of one hand, which lasted 10 minutes. At age 35, she presented with transient left brachiofacial hemiplegia (lasting approximately four hours) associated with somatosensory and aphasic auras. From age 45 onwards, there was a progressive reduction in the frequency with apparent remission in her fifties. Brain MRI was normal**.**

At age 22 and while 12 weeks pregnant, the other twin experienced an episode of tingling of the right hand and forearm without headache that lasted for 30 minutes. During puerperium, she presented with episodes of scintillating scotoma followed by bilateral numbness of the face, lips, cheek and tongue. Numbness was followed by either left unilateral, right unilateral or bilateral headache. At age 26, she experienced episodes of paraesthesia and numbness of both hands up to the elbows. Four years later, she presented with aphasia towards the end of the somatosensory aura, irrespective of the side of the other aura symptoms or headache. Occasionally, these episodes would resolve during sleep; frequently, they would respond to intravenous salicylates and metoclopramide. She currently experiences six episodes per year. The patient never spontaneously reported neuropsychological aura symptoms and denied their presence when questioned. Brain MRI was also normal**.**

### Other clinical features

We highlight three additional clinical features: late-life generalised tonic-clonic seizures (patriarch); *grand mal* seizures and death from *status epilepticus* at age 14 (II:8); remission of FHM episodes following subarachnoid haemorrhage and surgical repair of ruptured anterior communicant artery and anterior choroidal artery aneurysms at age 47 (II:15).

## Conclusions

The family described herein presents a high incidence of migraine (with and without aura), especially in the second generation. The transmission pattern is consistent with autosomal dominant inheritance. Several individuals (II:12, II:15, II:18, III:3) experienced motor aura and had relatives with identical phenotypes, fulfilling the criteria for FHM. However, their transmitting parents experienced only MoA (I:1) or migraine with visual aura (II:4). In this kindred, the phenotypes were variable between siblings and generations; nonetheless, all patients also experienced episodes of MoA. It is well known that compared with the general population, FHM probands and their first degree relatives have a significantly increased risk of migraine with typical aura. These results suggest that the genetic abnormality causing FHM may also cause MWA [14]. However, FHM is not a risk factor for MoA [14].

The monozygotic twin pair presents an intriguing pair of phenotypes. The twins shared many clinical similarities but also exhibited major phenotypic differences, despite having the same genetic background and living in the same environment until their teenage years. The temporal profile of migraine progression was drastically different. For one of the twins, the age of onset was menarche (11 years of age), with frequent episodes and apparent remission before menopause. The other twin had her first episode as an adult (age 22), with severe but rare episodes that continue to persist. The twins presented with identical visual and aphasic auras, as well as bilateral somatosensory auras, a rare phenomenon in FHM that was not experienced by any other family members. While the twins shared many similarities, one also experienced motor and neuropsychological auras while the other did not ever experience either type. Forced thinking did not occur in any other family members. Symptoms of this type are usually associated with frontal or temporal lobe epilepsy [15-17]. By way of analogy, the aura sequence suggests that spreading depression may reach the deep brain structures (deep left frontal operculum and limbic system). We can consider this aura to be consistent with prior reports of an association between epilepsy and migraine, particularly all three types of FHM. Interestingly, two deceased family members (I:1, II:8) had epilepsy. It remains unclear why this phenomenon is so rare, not even appearing in monozygotic twins.

Some individuals experienced bilateral somatosensory symptoms or dizziness but failed to fulfil the diagnostic criteria for migraine with brainstem aura due to the co-existence of motor aura. However, the presence of dizziness in FHM has been previously described; several authors have claimed that migraine with brainstem aura is a subtype of FHM [18,19].

In this family, there was one curious case of FHM with remission after surgery for a cerebral aneurysm. This outcome may have resulted from age-related remission or a biological response to a brain insult. This hypothesis is attractive when considering the role of non-genetic factors. This speculation may be interesting in the near future, when the pathophysiological basis of invasive therapies for migraine will be under discussion [20].

In this family, the wide array of symptoms, severity, age of onset and frequency (from scarce attacks to over 200 episodes across a lifetime) suggests that genetic factors do not have an exclusive role in the clinical expression. It is possible that the manifestation of the most severe phenotype is due to an unlikely combination of genetic and non-genetic factors (endogenous or environmental factors, which may be precipitating or modifying the phenotype).

It has been repeatedly argued that MoA and FHM are at the extreme ends of the proposed spectrum of migraine [1]. However, studies devoted to testing for the presence of FHM mutations in additional migraine types have yielded negative results [1]. In this family, we did not observe any mutations in the known FHM genes, although an unknown mutation may exist. However, the majority of individuals did not experience motor auras, and furthermore, major phenotypic differences were observed between a set of monozygotic twins. We may speculate that this family presents a polygenic multifactorial form of migraine; it is known that given the right environmental context, some individuals can develop motor aura despite not having a specific FHM genotype. On the other hand, we must not forget that families with known FHM mutations may present a complex phenotypic spectrum without motor aura symptoms [21].

Finally, we note that severe sensitive aura may be misinterpreted as motor aura. It is well known that sensitive auras may be perceived as paralysis (when they severely affect limbs) or aphasia (if orofacial). Nevertheless, the testimony of relatives makes this possibility more remote, although not impossible. Unfortunately, the short duration of the symptoms prevented the patients from reaching the hospital in time for an examination during the aura.

There is a clear genetic role in the determination of the migraine phenotype. However, genotype is not the only factor involved in the manifestation of clinical phenomena; the variable influence of non-genetic factors (inductive, favouring, modifying) is presumed. The boundaries separating different types of migraine are highly variable. In certain families, FHM is a monogenic disorder distinct from migraine with typical aura. In other families, such as this one, we propose that hemiplegic aura is a more severe and complex form of typical aura. Population-based epidemiological studies and molecular biology studies are likely to shed light on this recurrent dilemma.

## Consent

Written informed consent was obtained from the patients for publication of this case report. A copy of the written consent form is available for review by the Editor-in-Chief of this journal.

## Abbreviations

FHM: Familial hemiplegic migraine; MWA: Migraine with aura; MoA: Migraine without aura.

## Competing interests

The authors declare that they have no competing interests.

## Authors’ contributions

JB conceived of the study and its coordination, participated in all clinical observations, and helped to draft the manuscript; RB participated in clinical observations and helped to draft the manuscript; AFB carried out the molecular genetic studies; JD^s^ participated in clinical observations and helped to draft the manuscript; JD^o^ participated in clinical observations and helped to draft the manuscript; CR carried out magnetic resonance imaging studies; CL carried out the molecular genetic studies; helped to draft the manuscript; JS conceived the molecular genetic studies; IA conceived and carried out the molecular genetic studies; helped to draft the manuscript; JP-M participated in clinical observations and helped to draft the manuscript. All authors read and approved the final manuscript.
